# Adjuvant chemoradiotherapy for positive hepatic ductal margin on cholangiocarcinoma

**DOI:** 10.1002/ags3.12345

**Published:** 2020-05-15

**Authors:** Teiichi Sugiura, Katsuhiko Uesaka, Yukiyasu Okamura, Takaaki Ito, Yusuke Yamamoto, Ryo Ashida, Katsuhisa Ohgi, Hirofumi Asakura, Akiko Todaka, Akira Fukutomi

**Affiliations:** ^1^ Division of Hepato‐Biliary‐Pancreatic Surgery Shizuoka Cancer Center Shizuoka Japan; ^2^ Radiation and Proton Therapy Center Shizuoka Cancer Center Shizuoka Japan; ^3^ Division of Gastrointestinal Oncology Shizuoka Cancer Center Shizuoka Japan

**Keywords:** adjuvant chemoradiotherapy, cholangiocarcinoma, positive ductal margin, stump recurrence, survival

## Abstract

**Aim:**

This study evaluated the effects of postoperative adjuvant chemoradiotherapy (A‐CRT) for positive hepatic ductal margin (HM+) in extrahepatic cholangiocarcinoma (EHCC).

**Methods:**

Patients with EHCC who underwent surgical resection between 2002 and 2014 were included in this retrospective study. For patients with HM+, A‐CRT was conducted. The clinical effect of A‐CRT for HM+ on the survival and recurrence and prognostic factors of EHCC was reviewed.

**Results:**

Among 340 patients, the hepatic ductal margin was negative in 296 and positive in 44. Of the 44 patients with HM+, 22 received postoperative A‐CRT, and 22 did not. Hepatic stump recurrence occurred in 19 patients. The incidence was significantly higher in patients with HM+ (20%, 9/44) than in those with negative hepatic ductal margin (HM−) (3%, 10/296) (*P* < .001). Among the patients with HM+, the incidence was almost identical between the patients with and without A‐CRT: 23% (5/22) in HM+/CRT− and 18% (4/22) in HM+/CRT+ patients (*P* = .999). The median survival time was 49 months in HM−, 43 months in HM+/CRT−, and 49 months in HM+/CRT+ patients. The differences were not significant among the groups. A multivariate analysis revealed CA 19‐9 ≥ 300 U/mL, combined vascular resection, histologic grade G2/G3, and lymph node metastasis to be significant prognostic factors. However, the performance of postoperative A‐CRT did not contribute to prolonging survival.

**Conclusion:**

A‐CRT for HM+ in patients with EHCC did not affect the survival or stump recurrence.

## Graphical abstract

Adjuvant chemoradiotherapy was delivered for positive hepatic ductal margin in cholangiocarcinoma. However, this treatment did not affect the survival or stump recurrence.

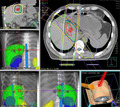

## 1 INTRODUCTION

Microscopically positive resection margins have been identified as one of the most important risk factors for recurrence of extrahepatic cholangiocarcinoma (EHCC) after radical surgery in previous studies.^1^, ^2^, ^3^ Therefore, extensive surgical procedures, including major hepatic resection for hilar cholangiocarcinoma, pancreatoduodenectomy for distal cholangiocarcinoma, and hepatopancreatoduodenectomy for diffusely spreading cholangiocarcinoma are strongly advocated. Local recurrence developed in 8%‐13% of patients who underwent resection for cholangiocarcinoma.^1^, ^2^, ^3^, ^4^, ^5^ Stratified by hepatic ductal margin (HM) status, the incidence of stump recurrence was 5%‐9% in patients with negative hepatic ductal margin (HM−), 8%‐30% in patients with positive hepatic ductal margin (HM+) with carcinoma in situ (CIS), and 24%‐50% in patients who had HM+ with invasive carcinoma, although the definition of stump recurrence differs among studies.^1^, ^2^, ^3^, ^4^, ^5^ Local recurrence originates from remnant or seeding cancer cells at surgical sites. Stump recurrence results in liver failure due to not only obstructive jaundice but also repeated (uncontrollable) cholangitis.

In cases with microscopically positive resection margins after aggressive surgery, adjuvant local treatment is required for the elimination of residual tumor to improve the survival. Several studies have reported that adjuvant chemoradiotherapy (A‐CRT) may improve the survival in patients who had R1 resection.^6^, ^7^ However, these studies included patients receiving adjuvant radiotherapy alone, brachytherapy, and intraoperative radiotherapy, and the survival benefits of adjuvant concurrent CRT after R1 resection have not fully been investigated.

In the authors’ institution, postoperative A‐CRT targeting the biliary stump at the hepatic hilum in patients with HM+ has been conducted aiming to reduce stump recurrence. The aim of this study was to review the effects of postoperative CRT for HM+ in EHCC.

## 2 METHODS

Data from consecutive patients with EHCC treated at the authors’ institution between 2002 and 2014 were obtained from a prospectively collected database and reviewed retrospectively. Patients with intrahepatic cholangiocarcinoma involving the hepatic hilum, distant metastasis, and in‐hospital mortality were excluded from the analysis. The incidence of stump recurrence, postoperative survival, and prognostic factors was evaluated by referencing the HM status or CRT practice. TNM classifications were determined according to the UICC system, 8th edition. The study was approved by the institutional review board.

### 2.1 CRT for HM+

Our standard of treatment for EHCC has been surgery alone, regardless of the tumor stage. CRT was targeted at patients with HM+. During this study period, HM+ was defined as a positive hepatic ductal margin with both invasive carcinoma and carcinoma in situ (CIS). After *providing some information* —as (a) positive hepatic ductal margin is correlated with stump recurrence, and (b) *A‐CRT is intended to prevent stump recurrence at the hepatic ductal stump*; (c) however, there has been no prospective study of A‐CRT for a positive ductal margin, and (d) several retrospective studies have revealed controversial results —*the decision* *on whether or not to receive A‐CRT was left to the patient*. However, A‐CRT was not intended for cases with a positive distal ductal margin. If recurrence at the distal margin stump alone occurs in the future, then additional pancreatoduodenectomy is considered to be a treatment option. In addition, the radiation field did not cover the regional lymph nodal basin because lymph node dissection was systematically performed in order to not leave remnant lymph nodes.

**Figure 1:**
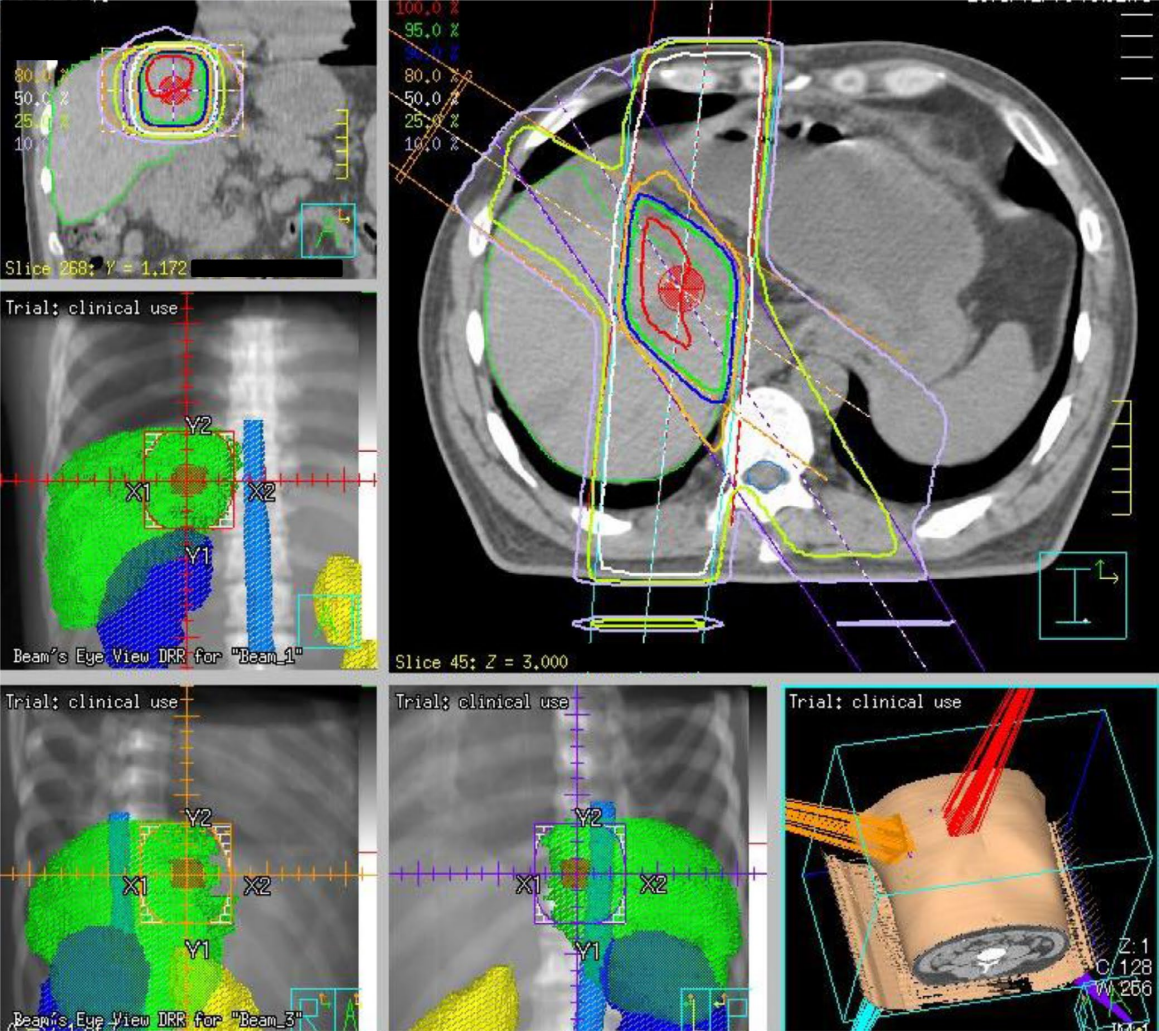
Simulation of radiotherapy. Radiation was targeted at the biliary stump at the hepatic hilum

Three‐dimensional conformal radiotherapy was planned to deliver a total of 50.4 Gy at 1.8 Gy per fraction, 5 days a week, except for in two patients who received 50 Gy at 2.0 Gy per fraction. Treatment was delivered using a linear accelerator with a 6‐ to 18‐MV photon beam. The treatment planning was based on computed tomography (CT) scans obtained in the treatment position. The clinical target volume (CTV) was defined by the operating surgeon as the area around the stump where the presence of microscopic residual tumor was considered likely based on intraoperative findings and postoperative imaging. The regional lymph nodes were not intended to be included in the CTV. The radiation fields were designed to cover the CTV with an adequate margin (i.e., 1‐2 cm in the axial direction and 1.5‐2.5 cm in the craniocaudal direction). Multifield arrangements with two to four beams or a conformal arc technique were used. Figure 1 shows an example of the radiotherapy planning. The concurrent chemotherapy regimen was 5‐FU or S‐1. Intravenous administration of 5‐FU (200 mg/m^2^/24 h for 7 days over weeks 1‐6) was concurrently conducted. S‐1 at an oral dose of 30, 40, or 50 mg according to the body surface area was orally provided twice a day on the same day as radiation. Maintenance chemotherapy was performed in all patients after the completion of concurrent radiotherapy up to 6 months after the initiation of CRT; 5‐FU at 220 mg/m^2^/24 h or S‐1 at 30‐50 mg was administered twice a day for 4 weeks with a 2‐week break.

### 2.2 Follow‐up and definition of recurrence

Postoperative adjuvant chemotherapy was not routinely performed except for in patients attempting CRT due to HM+. A few patients with HM‐ were enrolled in the clinical trial and received adjuvant chemotherapy with Gemcitabine.^8^ Within the first 3 years after resection, follow‐up examinations, including physical examinations, laboratory tests, assessment of tumor markers, and CT scans were performed at 3‐month intervals. At 3 years after surgery, if patients showed no signs of recurrence, they were followed‐up at 6‐month intervals.

The site of recurrence was confirmed based on radiologic or biopsy‐proven evidence. Stump recurrence was specifically defined as a local ill‐defined mass consistent with the hepatic stump accompanied by intrahepatic bile duct dilation and/or positive findings of positron‐emission tomography, increases in tumor markers, and increases in size over time on serial imaging to detect disease progression.

### 2.3 Statistical analyses

All statistical analyses were performed using the SPSS software program (version 25.0; SPSS, Inc). Continuous variables were expressed as median values with the range and dichotomized by referring to the minimum *P*‐values for the survival analysis. The chi‐square test or Fisher's exact test was performed for categorical variables where appropriate. A multivariate regression analysis of factors with a *P*‐value of < .10 on univariate analyses (log‐rank test) was performed using the Cox proportional hazard model. A *P*‐value of < .05 was considered to be statistically significant.

## 3 RESULTS

A total of 340 patients who underwent surgical resection for EHCC between 2002 and 2014 were included in this study. The surgical procedures were major hepatectomy with caudate lobectomy in 140 patients, pancreatoduodenectomy in 140 patients, and hepatopancreatoduodenectomy in 60 patients.

**Figure 2:**
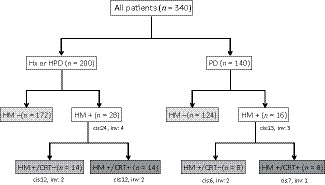
Schematic illustration of the hepatic ductal margin status and adjuvant chemoradiotherapy

The hepatic ductal margin status is presented in Figure 2. Two hundred and ninety‐six HM− and 44 HM+ were detected; CIS was found in 37 patients and invasive carcinoma in seven patients. Among 44 patients with HM+, 22 received A‐CRT (20 received 5‐FU, and two received S‐1) and 22 were followed without any additional treatment. Table 1 shows the demographics of the patients. There were no significant differences among the three groups regarding patients’ background characteristics, surgical procedure, or pathologic findings. All patients could receive the planned cycle of A‐CRT. Grade 3 adverse events (febrile neutropenia and nausea) occurred in two patients. After the completion of A‐CRT, no patients developed benign anastomotic strictures caused by radiation.

**Table 1:** Characteristics of patients according to the hepatic ductal margin and treatment status

	HM−	HM+/CRT‐	HM+/CRT+	*P*
	(n = 296)	(n = 22)	(n = 22)	
Age (y.o)	70 (37‐85)	77 (65‐85)	68 (40‐77)	.825
Sex				
Male	221 (75%)	17 (77%)	17 (77%)	.999
Female	75 (25%)	5 (23%)	5 (23%)	
Location				
Perihilar	163 (55%)	13 (59%)	10 (45%)	.634
Distal	133 (45%)	9 (41%)	12 (55%)	
CA19‐9 (U/mL)	61 (2‐27 286)	37 (3‐25 411)	72 (2‐4369)	.641
Surgery				
PD	124 (42%)	8 (36%)	8 (36%)	.785
Hx‐BDR/HPD	172 (58%)	14 (64%)	14 (64%)	
Hepatic artery resection				
No	262 (88%)	19 (86%)	20 (91%)	.894
Yes	34 (12%)	3 (14%)	2 (9%)	
Portal vein resection				
No	256 (86%)	21 (95%)	18 (82%)	.380
Yes	40 (14%)	1 (5%)	4 (18%)	
Histological grade^a^				
G1	119 (40%)	10 (45%)	13 (59%)	.209
G2/G3	177 (60%)	12 (55%)	9 (41%)	
T status				
pT1‐2	113 (38%)	11 (50%)	4 (18%)	.081
pT3‐4	183 (62%)	11 (50%)	18(82%)	
N status				
pN0	183 (62%)	12 (55%)	13 (59%)	.779
pN1/2	113 (38%)	10 (45%)	9 (41%)	

Note

Data are presented as mean and ranges of continuous variables, and as number and percentage for categorized variables.

Abbreviations: CA 19‐9, carbohydrate antigen 19‐9; CRT, chemoradiotherapy; HM, hepatic ductal margin; HPD, hepatopancreatoduodenectomy; Hx‐BDR, major hepatectomy with bile duct resection; PD, pancreatoduodenectomy.

^a^ UICC classification.

One hundred and eighty‐eight patients developed recurrence. Stump recurrence occurred in 19 patients. The incidence of stump recurrence in HM+ patients (20%, 9/44) was significantly higher than that in HM‐ patients (3%, 10/296) (*P* < .001). Among the HM+ patients, the incidence was identical in patients with and without A‐CRT (HM+/CRT−, 23% (5/22); HM+/CRT+, 18% (4/22), *P* = .999). Next, these patients were divided according to the degree of HM+. In 37 patients with HM+ with CIS, the incidence was almost identical, regardless of A‐CRT (HM+/CRT−, 11% (2/18); HM+/CRT+, 11% (2/19), *P* = .999). In seven patients with HM+ with invasive carcinoma, the incidence of stump recurrence was also almost the same, regardless of A‐CRT (HM+/CRT−, 75% (3/4); HM+/CRT+, 67% (2/3), *P* = .999). The median interval between surgery and the development of stump recurrence was 31 months in HM− patients, 43 months in HM+/CRT− patients, and 40 months in HM+/CRT+ patients. Other recurrence sites were the liver, lymph nodes, local area, peritoneum, and other hematologic metastases (lung, bone, etc.) (Table 2 **)**. The incidence of each recurrence site was identical. Of the 19 patients with stump recurrence, isolated stump recurrence was detected in nine, including five with HM−, 2 with HM+/CRT−, and two with HM+/CRT+. The other 10 patients had recurrence at multiple sites, including the liver in four, peritoneum in four, lung in four, lymph node in two, and local in two (overlapped in patients). After the detection of stump recurrence, five patients were treated with gemcitabine, three patients were treated with gemcitabine and cisplatin, two patients were treated with S‐1, and nine patients received the best supportive care. Aside from these treatments, nine patients underwent percutaneous transhepatic biliary drainage.

**Table 2:** Association between hepatic ductal margin and treatment status, and recurrence site

	HM‐	HM+/CRT‐	HM+/CRT+
	(n = 296)	(n = 22)	(n = 22)
Hepatic ductal stump	10 (3%)	5 (23%)	4 (18%)
Liver	67 (23%)	4 (18%)	4 (18%)
Lymph nodes	45 (15%)	4 (18%)	5 (23%)
Local	23 (8%)	3 (14%)	2 (9%)
Peritoneum	39 (13%)	3 (14%)	4 (18%)
Other distant^a^	24 (8%)	3 (14%)	2 (9%)

Note

Data are presented as number and percentage for categorized variables.

^a^ Distant metastasis other than liver and peritoneum.

**Figure 3:**
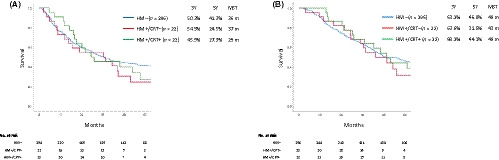
The disease‐free (A) and overall (B) survival according to the hepatic ductal margin and treatment status. A, *P* = .192 (HM− vs HM+/CRT−); *P* = .378 (HM+/CRT− vs HM+/CRT+); *P* = .754 (HM− vs HM+/CRT+). B, *P* = .281 (HM− vs HM+/CRT−); *P* = .468 (HM+/CRT− vs HM+/CRT+); *P* = .783 (HM− vs HM+/CRT+)

The disease‐free survival (DFS) stratified according to the margin and treatment status was nearly the same among the groups, with a median survival time (MST) of 36 months in patients with HM−, 37 months in those with HM+/CRT−, and 29 months in those with HM+/CRT+ (Figure 3A). The overall survival (OS) was also nearly the same among the groups, with an MST of 49 months in patients with HM−, 43 months in those with HM+/CRT−, and 49 months in those with HM+/CRT+ (Figure 3B).

Table 3 shows the prognostic factors for EHCC. A CA19‐9 value ≥300 U/mL, combined vascular resection, histological grade G2/G3, and the presence of lymph node metastases were shown to be significant prognostic factors. However, the performance of postoperative A‐CRT did not help prolong the survival.

**Table 3:** Prognostic factors for extrahepatic cholangiocarcinoma

	n	Univariate analysis		Multivariate analysis	
		MST	*P*	HR (95% CI)	*P*
Location					
Perihilar	154	60	.136		
Distal	186	45			
CA19‐9 (U/mL)					
<300	279	57	<.001	1	
≥300	61	34		1.67 (1.20‐2.30)	.002
Vascular resection^a^					
No	269	60	<.001	1	
Yes	71	36		1.78 (1.19‐2.63)	.004
Histological grade^b^					
G1	142	82	<.001	1	
G2/G3	198	42		1.72 (1.27‐2.32)	<.001
T status^b^					
pT1‐2	128	78	.003	1	
pT3‐4	212	43		1.17 (0.85‐1.61)	1.611
N status^b^					
pN0	208	80	<.001	1	
pN1/2	132	36		1.81 (1.34‐2.42)	<.001
Lymphvascular invasion					
No	82	87	.001	1	
Yes	258	44		0.95 (0.65‐1.46)	.909
Perineural invasion					
No	65	162	<.001	1	
Yes	275	45		1.46 (0.93‐2.29)	.1
Portal vein invasion					
No	292	53	.034	1	
Yes	48	36		0.83 (0.52‐1.34)	.459
Hepatic artery invasion					
No	304	53	.044	1	
Yes	36	36		0.89 (0.57‐1.41)	.632
Adjuvant CRT					
No	318	49	.836		
Yes	22	49			

Abbreviations: CA 19‐9, carbohydrate antigen 19‐9; CI, confidence interval; CRT, chemoradiotherapy; HR, hazard ratio; MST, median survival time.

^a^ Hepatic artery and/or portal vein resection.

^b^ UICC classification.

**Figure 4:**
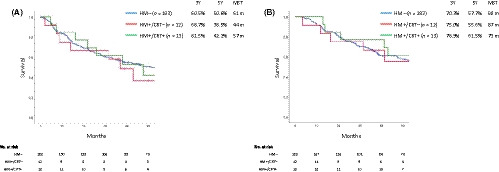
The disease‐free (A) and overall (B) survival according to the hepatic ductal margin and treatment status in patients without lymph node metastasis. A, *P* = .342 (HM− vs HM+/CRT−); *P* = .429 (HM+/CRT− vs HM+/CRT+); *P* = .969 (HM− vs HM+/CRT+). B, *P* = .504 (HM− vs HM+/CRT−); *P* = .462 (HM+/CRT− vs HM+/CRT+); *P* = .874 (HM− vs HM+/CRT+)

**Figure 5:**
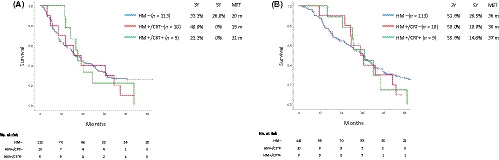
The disease‐free (A) and overall (B) survival according to the hepatic ductal margin and treatment status in patients with lymph node metastasis. A, *P* = .631 (HM− vs HM+/CRT−); *P* = .739 (HM+/CRT− vs HM+/CRT+); *P* = .818 (HM− vs HM+/CRT+). B, *P* = .666 (HM− vs HM+/CRT−); *P* = .942 (HM+/CRT− vs HM+/CRT+); *P* = .615 (HM− vs HM+/CRT+)

Because lymph node metastasis was the strongest prognostic factor in EHCC, a sub‐analysis of the pN0 and pN1/2 groups was performed. In patients with pN0, the DFS was nearly the same among the groups, with an MST of 61 months in HM− patients, 44 months in HM+/CRT− patients, and 57 months in HM+/CRT+ patients (Figure 4A). The OS was also nearly the same among the groups, with an MST of 80 months in HM− patients, 87 months in HM+/CRT− patients, and 73 months in HM+/CRT+ patients (Figure 4B). In patients with pN1/2, the DFS was nearly the same among the groups, with an MST of 20 months in HM− patients, 19 months in HM+/CRT− patients, and 21 months in HM+/CRT+ patients (Figure 5A). The OS was also nearly the same among the groups, with an MST of 36 months in HM− patients, 36 months in HM+/CRT− patients, and 37 months in HM+/CRT + patients (Figure 5B).

## 4 DISCUSSION

Complete resection is the mainstay treatment for patients with EHCC.^9^ However, surgeons occasionally face the issue of a positive resection margin, especially at the hepatic ductal stump.^3^, ^10^ Even if complete resection is achieved, one of the most common patterns of failure for EHCC is locoregional recurrence.^11^, ^12^ To control stump recurrence and prolong the prognosis, A‐CRT was administered in these patients. However, we failed to reveal any marked benefit of A‐CRT. A‐CRT for HM+ was not effective for improving the survival or stump recurrence. Stratified by the degree of HM+, the stump recurrence rate in patients with HM+ with CIS was nearly the same. Although few patients with HM+ had invasive carcinoma, two of the three who received A‐CRT developed stump recurrence. In that sense, A‐CRT does not seem to be effective for HM+ with CIS or invasive carcinoma.

Several previous studies evaluated the effect of A‐CRT on reducing the locoregional recurrence for cholangiocarcinoma. There have been conflicting results regarding the effect of adjuvant radiotherapy with and without chemotherapy after curative surgical resection.^6^, ^13^, ^14^, ^15^ Some studies have shown that postoperative adjuvant radiotherapy has no influence on survival^13^, ^14^. In contrast, others have reported survival advantages of CRT.^6^, ^15^, ^16^, ^17^ Two recent meta‐analyses found that adjuvant therapy including radiotherapy for cholangiocarcinoma decreased the risk of death compared to surgery alone, especially in cases with lymph node metastases or a positive surgical margin.^18^, ^19^ However, another meta‐analysis by Zhu^20^ revealed that CRT was not effective for margin‐positive disease. In all of these studies, radiation was delivered to the tumor bed and regional lymph nodes. In our series, radiation was strictly targeted at the hepatic ductal stump. This is therefore the first report to evaluate the effect of CRT on controlling stump recurrence.

In the present study, a CA19‐9 value ≥300 U/mL, combined vascular resection, histological grade G2/G3, and lymph node metastases were found to be significant prognostic factors. In particular, a higher CA19‐9 value, lymph node metastasis, and poor differentiation influenced the development of distant metastases.^12^, ^21^, ^22^ In the present study, 10 of 19 patients with stump recurrence also had multiple‐site recurrence, especially distant metastasis. All of these patients had at least one prognostic factor. In contrast, only two of nine patients with isolated stump recurrence had these prognostic factors. In patients at a high risk of distant metastases, the efficacy of CRT for local control seems to be low, and more powerful systemic chemotherapy regimens are needed.

Chemotherapy for advanced cholangiocarcinoma has been gradually established since 2005.^23^, ^24^ The findings of three randomized studies in an adjuvant setting have been published in that time.^8^, ^25^, ^26^ However, none of those studies demonstrated the effectiveness of adjuvant chemotherapy in an intention‐to‐treat analysis.^8^, ^25^, ^26^ Only capecitabine chemotherapy, which extended the survival in a per‐protocol analysis,^26^ was moderately recommended in the American Society of Clinical Oncology guideline.^27^ Therefore, no adjuvant chemotherapy has yet been strongly recommended based on evidence.

Whether or not CIS should be included in HM+ is controversial. In the present study, most of the residual cancer in HM+ cases were CIS. This may be due to the advent of precise preoperative imaging diagnoses and aggressive surgery, such as major hepatectomy. CIS, defined as ‘‘non‐invasive cancer’’, is often present near the main tumor.^28^ Some authors have reported that the survival rate of patients with residual CIS at the ductal stump after surgery is comparable to that for patients with R0 resection.^5^, ^10^ However, stump recurrence from residual CIS foci develops occasionally within 5‐10 years.^3^, ^5^ In addition, residual CIS increases the incidence of stump recurrence and reduces the survival in patients with early‐stage cholangiocarcinoma.^3^ Therefore, we had considered that additional treatment targeting ductal stump would be necessary, especially in patients with early‐stage cholangiocarcinoma.

In the current series, the CRT regimen was relatively outdated. 5‐FU was mainly used because this treatment started in the early 2000s. In 2005, chemotherapy for advanced cholangiocarcinoma was established, and the efficacy of GEM,^29^ oral fluoropyrimidine (S‐1),^30^ and GEM + Cisplatin^23^ was proven. Although the present study failed to demonstrate the control of the stump recurrence and survival, new CRT regimens using these agents are worth a try.

The major limitation of the present study is its retrospective nature and single‐institutional setting. The number of subjects was also not sufficient to draw broad interpretations. In particular, the number of stump recurrence events was low. A slight increase in the events can affect the statistical analyses. A longer follow‐up period is necessary, as some cases of stump recurrence develop more than 5 years later.

## 5 CONCLUSION

CRT for HM+ was not effective for improving survival or stump recurrence in patients with EHCC.

## DISCLOSURE

Conflict of Interest: Authors declare no conflict of interest for this article.
